# Cryptococcal Meningitis Presenting as New-Onset Seizures in an Immunocompetent Patient

**DOI:** 10.1177/2324709619861129

**Published:** 2019-07-27

**Authors:** Andrea Akyeampong, Nadia Solomon, Nicholas A. Boire, Aam A. Baqui

**Affiliations:** 1 St. George’s University, True Blue, Grenada; 2 The Brooklyn Hospital Center, Brooklyn, NY, USA

**Keywords:** cryptococcosis, diagnostic delay, infectious disease, pathology, seizure

## Abstract

This report describes a 30-year-old immunocompetent male with new-onset seizures, later found on imaging to have 2 enhancing lesions in the brain. The patient underwent a left parietal craniectomy with resection of one of the masses, which demonstrated focal areas of necrosis and many small cystic structures positive for periodic acid-Schiff and Gomori’s methenamine silver special stain. Numerous laboratory examinations, including HIV test, rapid plasma reagin, toxoplasma immunoglobulin G and immunoglobulin M, Lyme, cytomegalovirus, tuberculosis, cysticercosis, and *Echinococcus* serology, were all negative. Despite negative cerebrospinal fluid (CSF) culture and several negative CSF antigen tests, continued investigation, and follow-up, CSF antigen testing ultimately revealed *Cryptococcus* as the causative agent. In light of the mysterious and unusual presentation, the authors discuss potential infectious differential diagnoses in patients with atypical clinical presentation, laboratory tests, and surgical pathology.

## Introduction

Although seizures may be due to any number of causes, infections and their sequelae should always be considered when confronted with a case of new-onset seizures in an adult.^1^ The following report presents one such case, of a 30-year-old immunocompetent male with new-onset seizures, later found on imaging to have 2 enhancing lesions in the brain. The ensuing investigation, its findings and pitfalls, is described, and utilized to launch a discussion of potential infectious differential diagnoses in patients with atypical clinical presentation, laboratory tests, and surgical pathology.

## Case Report

**Figure 1.**
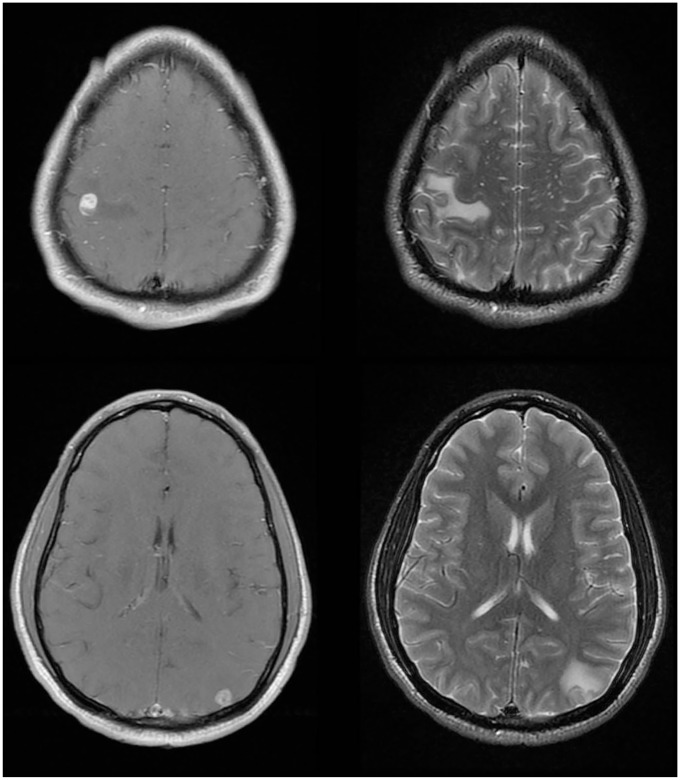
Magnetic resonance imaging of the brain demonstrates 2 enhancing masses, both associated with vasogenic edema.

**Figure 2.**
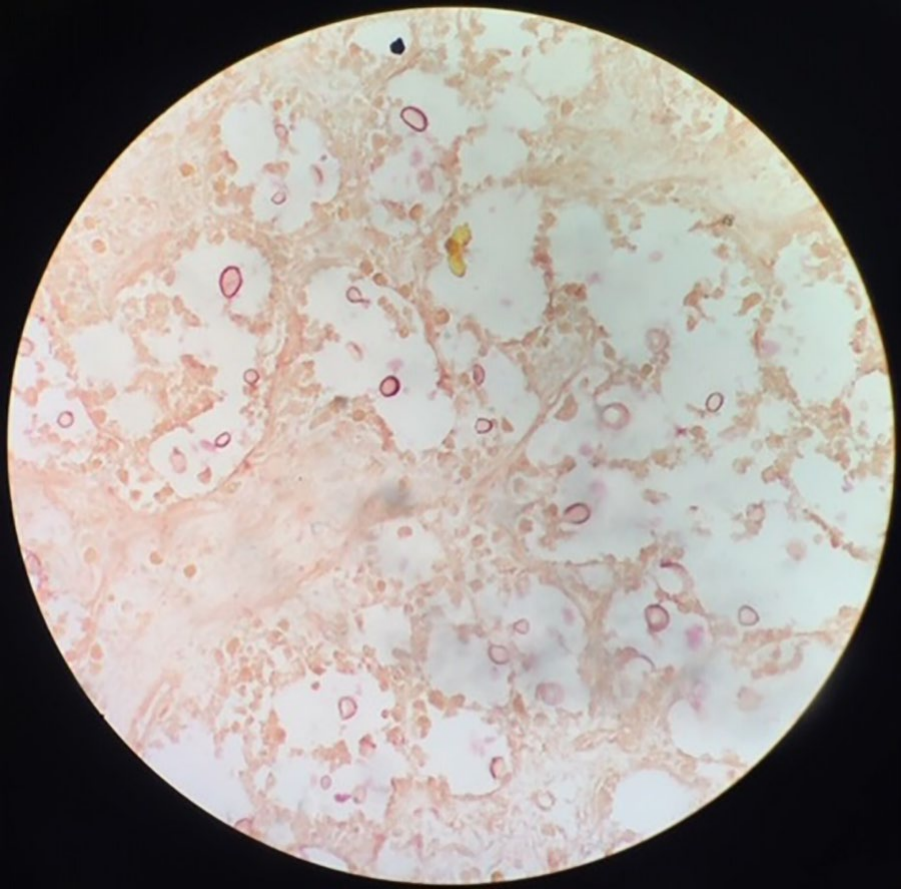
At low magnification (40×), mucicarmine special stain reveals small, positive-staining cystic structures.

A 30-year-old male presented with a witnessed episode of new-onset seizure: the patient reported suddenly feeling numb in his left third through fifth digits, followed by shaking of his left hand and a locking sensation, at which point he called for help. He then recalled waking up on the floor in a state of confusion regarding where he was and how he ended up there. He reported no presyncopal symptoms, incontinence, tongue-biting, or myalgias. The patient had no significant past medical or surgical history, but has a social history significant for marijuana use. The patient endorsed frequent travels to Mexico 5 years ago when he went to college in California, as well as travels to Puerto Rico 7 years ago. Computed tomography (CT) scan of head revealed areas of hypointensity in the right frontoparietal and left parieto-occipital lobes. Magnetic resonance imaging (MRI) with and without contrast demonstrated enhancing masses suspicious for metastasis versus abscesses associated with vasogenic edema, without evidence of midline shift (Figure 1). On day 6 of admission, the patient underwent a left parietal craniectomy with resection of the mass from left parieto-occipital lobe. Pathological examination of the mass revealed fragments of non-epithelialized fibrous cyst wall, with neutrophils, lymphocytes, plasma cells, and some eosinophils. Focal areas of necrosis were noted, and many small cystic structures were seen within the wall. Special stain showed that these small cysts were positive for periodic acid-Schiff, Gomori’s methenamine silver, and mucicarmine special stain (Figure 2), and negative for acid fast bacilli special stain. Pathology suggested these findings were consistent with the walls of *Echinococcus*/hydatid cyst. Numerous laboratory examinations, including HIV, rapid plasma reagin, toxoplasma immunoglobulin G and immunoglobulin M, Lyme, cytomegalovirus, tuberculosis, cysticercosis, and *Echinococcus* serology, were all negative. Cerebrospinal fluid (CSF) culture was negative, and several CSF *Cryptococcus* antigen tests were negative as well.

Ultimately, a second opinion of the surgical pathology noted granulomatous inflammation with abscess formation in association with microorganisms most consistent with *Cryptococcus*, and follow-up *Cryptococcus* antigen testing was found to be positive. The following discussion addresses the pathological and antigen test findings, and reviews each differential diagnosis and why it was considered.

## Discussion

*Cryptococcus* meningitis (CM) is a common cause of morbidity in immunocompromised individuals worldwide, and in rare cases, immunocompetent individuals may be affected as well.^2^ *Cryptococcus* infection occurs when fungal spores are inhaled and phagocytized by alveolar macrophages in the lung where they can cause pulmonary disease in immunocompromised patients.^3^ *Cryptococcus neoformans* and *C gattii* account for the majority of the disease burden, with *C neoformans* types A and D accounting for most infections in immunocompromised patients, with serotypes B and C accounting for most infections in immunocompetent patients.^3^ Clinical presentation can be highly variable, but may include headache, fever, neck pain, nausea, vomiting, light sensitivity, or altered mental status. Ten percent to 30% of HIV-negative patients with CM have no apparent underlying cause.^4^ Diagnosis is usually through cryptococcal antigen test and culture of CSF. Risk factors include AIDS, organ transplants, chronic corticosteroid use, cancer, and idiopathic CD4+ lymphocytopenia.^3^ Although neuroimaging is usually normal in CM, cryptococcomas, pseudocysts, and obstructing hydrocephalus can be seen.^5^ MRI is considered the most sensitive imaging modality, but can also be indistinguishable from other differentials including tuberculosis or metastatic malignancy.^6^

*Cryptococcus* can be either acapsular (lacking major capsular antigen) or hypocapsular (indistinguishable from wild type); capsular modifications can occur after prolonged in vitro growth or in vivo passaging, which can vary in different organs.^2^ Changes in capsular structure occur with passage of the organism through the blood-brain barrier.^2^ This can affect the host-pathogen interaction, thereby affecting diagnosis, and may in part explain why CSF cultures were initially negative in our patient.^2^ There have been similar cases of delayed diagnosis of CM due to negative CSF findings. In one case report, a patient initially considered immunocompetent developed CM and was later found to have been lymphocytopenic prior to and throughout the course of the disease; from this, investigators concluded that the patient may have had a subtle immunodeficiency, a possible risk factor for developing CM.^4^ Another study described a “post-zone phenomenon” in which excessive antigen relative to antibodies prevents antigen-antibody cross-linking (required for immunochromatographic detection) and leads to false-negative cryptococcal antigen testing.^7^ Overall, false-negative testing in an immunocompetent individual with CM creates difficulties for patient management due to the resultant delay in diagnosis.

The mysterious nature of this case led to formulation of a number of other differential diagnoses, among which several parasitic etiologies—including coenurosis, neurocysticercosis, and neurohydatidosis—were considered.

Coenurosis, a human zoonotic disease caused by larvae of the *Taenia* species (*T multiceps, T solium*, etc), is normally found in dogs.^8^ Humans accidentally ingest eggs, which release oncospores that penetrate the intestines, travel the bloodstream, and eventually lodge in the brain, spinal cord, and eyes. In the brain, oncospores causes inflammation coenorosis in the parenchyma. Clinical presentation includes headache, seizures, vomiting, and papilledema. Focal neurological deficits such as cranial nerve palsy and motor weakness are commonly seen.^8^ Definitive diagnosis is through removal of the cyst and polymerase chain reaction to identify the pathogen. On CT, cerebral coenurosis exhibits hypoattenuating spheroid lesions without central contrast enhancement. Edema is seen in late stages of the infection during cyst degeneration.^9^ Coenurosis is considered a diagnosis of exclusion after other pathogens are ruled out. There have been instances in literature where coenurosis mimics other diseases such as hydatid cysts.^10^

Neurocysticercosis is caused by the larval stage of the cestode *T solium*, and it is a major cause of new-onset adult seizures in developing countries.^11^ Because of our patient’s travel history, this differential was considered. Patients become infected on ingesting cysts in contaminated pork, with the scolex attaching to the intestines and maturing into a 2- to 4-mm tapeworm. Clinical presentation varies: in endemic countries, this pathogen is considered the great imitator, presenting similarly to many other pathogens.^11^ This can include tonic-clonic seizures, headache, focal neurological weakness, vomiting, and visual disturbance.^12^ Neuroimaging demonstrates cysts as single or multiple small, round, non-enhancing lesions with little to no edema. Calcifications representing degenerating cysticerci may also be seen. Diagnosis can be confirmed on imaging studies via demonstration of a cyst or several cysts containing a scolex, a nodule within the cyst.^12^

Neurohydatidosis is caused by infection of the brain with the tapeworm *Echinococcus granulosus*, which occurs when humans accidentally ingest the parasitic eggs. On MRI and CT, a cyst appears as a well-defined, smooth, homogenous oval or spherical mass isointense to CSF with a thin, low-intensity rim.^13,14^ Size may be variable, with the potential to reach up to 15 cm.^14^ Of importance, approximately 75% of patients with intracranial hydatid cysts are in the pediatric age range,^15^ suggesting that this was less likely to be the etiology in our adult patient. In addition, cerebral hydatid cysts tend to be large and solitary lacking surrounding vasogenic edema, in contrast to the multiple, small, edematous lesions seen in our patient.

## Conclusion

This case demonstrates that negative CSF cultures for *Cryptococcus* and negative CSF cryptococcal antigen tests cannot rule out cryptococcal infection. Delayed detection may be due to any number of proposed factors, including changes in capsular structure that occur as the organism passes through the blood-brain barrier, subtle immunodeficiency, or the “post-zone phenomenon.”^2,4,7^ Other infectious etiologies that should be considered in cases of new-onset seizures include coenurosis, neurocysticercosis, and neurohydatidosis.
